# Quality of care for hypertension in the United States

**DOI:** 10.1186/1471-2261-5-1

**Published:** 2005-01-07

**Authors:** Steven M Asch, Elizabeth A McGlynn, Liisa Hiatt, John Adams, Jennifer Hicks, Alison DeCristofaro, Roland Chen, Pablo LaPuerta, Eve A Kerr

**Affiliations:** 1 West LA VA, Mail Code 111G, 11301 Wilshire Bl, Los Angeles, CA 90073, USA; 2 RAND Health, 1776 Main Street, Santa Monica, CA 90407, USA; 3 Global Epidemiology and Outcomes Research, Pharmaceutical Research Institute, Bristol-Myers Squibb Company, PO Box 4000, Princeton, NJ 08543-4000, USA; 4 VA Center for Practice Management and Outcomes Research and the Department of Medicine, University of Michigan, Ann Arbor, (11H), 2215 Fuller Road, Ann Arbor, MI 48105, USA

## Abstract

**Background:**

Despite heavy recent emphasis on blood pressure (BP) control, many patients fail to meet widely accepted goals. While access and adherence to therapy certainly play a role, another potential explanation is poor quality of essential care processes (QC). Yet little is known about the relationship between QC and BP control.

**Methods:**

We assessed QC in 12 U.S. communities by reviewing the medical records of a randomly selected group of patients for the two years preceding our study. We included patients with either a diagnosis of hypertension or two visits with BPs of ≥140/90 in their medical records. We used 28 process indicators based on explicit evidence to assess QC. The indicators covered a broad spectrum of care and were developed through a modified Delphi method. We considered patients who received all indicated care to have optimal QC. We defined control of hypertension as BP < 140/90 in the most recent reading.

**Results:**

Of 1,953 hypertensive patients, only 57% received optimal care and 42% had controlled hypertension. Patients who had received optimal care were more likely to have their BP under control at the end of the study (45% vs. 35%, p = .0006). Patients were more likely to receive optimal care if they were over age 50 (76% vs. 63%, p < .0001), had diabetes (77% vs. 71%, p = .0038), coronary artery disease (87% vs. 69%, p < .0001), or hyperlipidemia (80% vs. 68%, p < .0001), and did not smoke (73% vs. 66%, p = .0005).

**Conclusions:**

Higher QC for hypertensive patients is associated with better BP control. Younger patients without cardiac risk factors are at greatest risk for poor care. Quality measurement systems like the one presented in this study can guide future quality improvement efforts.

## Background

Hypertension affects approximately 58 million Americans [1]. Lowering diastolic blood pressure (BP) by 10 mm Hg can reduce the number of strokes by as much as 56% and the incidence of coronary heart disease by 37% [2]. In addition, it has been shown that lowering systolic BP to 150 mm Hg decreases the incidence of all types of strokes [3]. Although treatment reduces mortality, morbidity and costs, nearly half of all people with hypertension go untreated and only 23% control their BP to the recommended level [4]. The high prevalence of uncontrolled hypertension is due in part to a lack of awareness: 32% of people with the disease do not know they have it [4]. However, over 40% of diagnosed hypertensive patients remain uncontrolled [4].

One potential explanation for uncontrolled hypertension is suboptimal quality of care. Although the U.S. Joint National Committee on Prevention, Detection, and Treatment of High Blood Pressure (JNC-VII) [5] has codified standards for clinical processes in hypertension in the United States, studies dating back to the 1970s have shown that many patients fail to receive this essential care [6-8]. On the other hand, many of these studies have not established a relationship between these care processes and BP control. One exception is a recent study of U.S. Veterans Administration patients in five facilities, which found a correlation between aggressive treatment and better-controlled BP [9]. Still, most studies examining the link between care processes and controlled hypertension generally have been confined to single delivery systems, a limited number of facilities, or a relatively small set of indicators of hypertensive quality. In a previous study, we examined general measures of hypertensive quality (including treatment, diagnosis, and follow-up indicators) and found that these care processes were associated with BP control in young women participating in a single health plan [10]. Studies with more generalizable target populations are lacking.

If deficits in process quality are indeed related to BP control, then which patients are failing to receive optimal care? The literature suggests that ethnic minorities and older patients are less likely to have controlled hypertension [4], but we do not know what clinical factors may be affecting patient care. For instance, physicians may be targeting higher-risk patients and administering better care to those with diabetes, coronary artery disease (CAD), and tobacco abuse. Likewise, providers may be delivering better care to older patients as they are also at higher risk, though limited evidence suggests the opposite is true [11,12].

We hypothesized that overall process quality for hypertensive care is associated with better BP control. We developed indicators of hypertensive care and determined whether patients had received the indicated care by reviewing medical records for a national sample of patients receiving care in a variety of settings. We also investigated whether patients with other cardiac risk factors received better hypertensive care and had better BP control.

## Methods

### Design and sampling

Our examination of hypertensive care was part of a larger study called the Community Quality Index Study (CQI), a cross-sectional study that assessed effectiveness of care for 32 different clinical conditions by examining medical records for patients in 12 randomly selected communities with populations greater than 200,000 (Boston, Cleveland, Greenville, Indianapolis, Lansing, Little Rock, Miami, Newark, Orange County, Phoenix, Seattle, and Syracuse) [13]. The methodology and overall results from CQI are presented elsewhere, and are summarized briefly here [14]. Between October 1998 and August 2000, study participants were selected by random digit dialing and asked permission to obtain copies of their medical records from all providers they had seen in the previous two years [14]. Of the 20,158 persons in the starting sample, we excluded 2,091 (10%) because they had moved out of the area, passed away, or become incapacitated in some manner that left them unable to participate in the study. Of the 17,937 adults who were eligible for the study, 74% (13,275) completed the telephone survey. Of the 12,412 participants who reported having at least one health care visit during the previous two years, 84% (10,404) agreed to medical record review and 7,528 signed consent forms. We obtained at least one record for 89% (6,712) of the respondents who consented, and we received 84% of the total records for which we had consent forms. Non-respondents were more likely to be female, older, and to have used health care services during the study period (p < .001).

### Development of quality indicators

We developed process indicators for hypertensive quality of care by reviewing the scientific literature and clinical practice guidelines [15] pertaining to hypertensive care [16,17]. The indicators represented clinical processes across the spectrum of hypertensive care and were based closely on JNC-VI [15]. A diverse expert panel of nine physicians reviewed the indicators and supporting evidence using a modified Delphi method. They rated each indicator's feasibility and validity using a 9-point Likert scale. Indicators were accepted if their median validity score was 7 or higher and their median feasibility score was 4 or higher. The final 28 quality-of-care indicators included 1 screening indicator, 14 diagnostic indicators, 8 treatment indicators, and 5 follow-up indicators. Eight of the indicators were supported by randomized controlled trials and 20 by JNC-VI or other expert guidelines. The full list of indicators is presented in Table 1.

**Table 1:** Performance of recommended hypertensive care indicators

**Indicator**	**Eligible (n)**	**Adherence to Indicators (%)**	**Standard Error (%)**
1. Systolic and diastolic blood pressure should be measured on patients otherwise presenting for care at least once each year.	1,953	72	1.0
2. All patients with average blood pressures of Stage 1 or greater as determined on at least 3 separate visits should have a diagnosis of hypertension documented in the record.	823	73	1.8
3. Patients with a new diagnosis of Stage 1–3 hypertension should have at least 3 or more measurements on separate visits with a mean SBP > 140 or a mean DBP > 90.	185	21	3.6
4. Medication and substance abuse: Personal history of tobacco abuse, alcohol abuse, or taking of medications that may cause hypertension;	183	35	5.0
5. Physical examination: examination of the fundi	199	14	2.8
6. Examination of heart sounds	199	71	4.1
7. Examination of abdomen for bruits	199	49	4.6
8. Examination of peripheral arterial pulses	199	32	4.1
9. Examination of neurologic system	199	35	4.6
10. Initial laboratory tests should include at least 5 of the following: Urinalysis;	241	30	4.3
11. Serum, plasma, or blood glucose;	241	65	4.2
12. Serum potassium;	241	59	4.4
13. Serum creatinine;	241	62	4.2
14. Serum cholesterol; or	241	58	4.2
15. Serum triglyceride.	241	60	4.4
16. First-line treatment for patients in risk group HN-A or HN-B, is lifestyle modification. The medical record should indicate counseling for at least 1 of the following interventions prior to initiating pharmacotherapy:	27	31	10.2
- weight reduction if obese;			
- increased physical activity if sedentary; or			
- low sodium diet.			
17. First-line treatment for patients with Stage 1A hypertension, is lifestyle modification. The medical record should indicate counseling for at least 1 of the following interventions prior to initiating pharmacotherapy:	25	25	10.4
- weight reduction if obese;			
- increased physical activity if sedentary; or			
- low sodium diet.			
18. Treatment for Stage 1B and 1C, and Stages 2 and 3 hypertension should include lifestyle modification. The medical record should indicate counseling for at least 1 of the following interventions:	149	40	5.3
- weight reduction if obese;			
- increased physical activity if sedentary; or			
- low sodium diet.			
19. Stage 1B hypertensives whose blood pressure remains Stage 1 after 6 months of lifestyle modification recommendation should be offered pharmacotherapy.	113	20	4.5
20. Stage 1A hypertensives whose blood pressure remains Stage 1 after 12 months of lifestyle modification recommendation should be offered pharmacotherapy.	22	14	7.6
21. Patients in any risk group with Stage 2–3 hypertension should be offered pharmacotherapy.	359	64	3.4
22. Patients in Risk group HN-C should be offered pharmacotherapy.	277	67	3.9
23. Patients in Risk group C with stage 1 hypertension should be offered pharmacotherapy.	332	75	2.9
24. Hypertensive patients should visit the provider at least once each year.	1,681	94	0.7
25. Newly diagnosed Stage 1 patients should be evaluated by the provider within 4 months of their initial visit.	111	76	5.1
26. Newly diagnosed Stage 2 patients should be evaluated by the provider within 2 months of their initial visit.	56	66	7.6
27. Newly diagnosed Stage 3 patients should be evaluated by the provider within 2 weeks of their initial visit.	18	33	12.9
28. Hypertensive patients with consistent average SBP > 140 or DBP > 90 over 6 months should have one of the following interventions recorded in the medical record:	853	77	1.8
- Change in dose or regimen of antihypertensives; or			
- Repeated education regarding lifestyle modifications.			
Overall		72	1.0

**Explanation of staging system used in Table 1 **Risk group A indicates no CAD risk factors or target organ damage or CAD. Risk group B indicates CAD risk factors, but no target organ damage or CAD or DM. Risk group C indicates target organ damage, DM or CAD. HN high-normal indicates 130–139 or 85–89. Stage 1 hypertension indicates 140–159 or 90–99. Stage 2 hypertension indicates 160–179 or 100–109. Stage 3 hypertension indicates ≥180 or ≥110.

### Data collection

After a two-week training course, twenty nurse abstractors used a computer-based abstraction tool with branching logic to abstract from the medical records we collected the following data for the two-year study period: a diagnosis of hypertension; a diagnosis of co-morbid disease, including CAD, diabetes, and hyperlipidemia; BP readings; laboratory results, including serum creatinine, cholesterol, trigylcerides, sodium, potassium, and urinalyses; prescriptions for anti-hypertensive agents; and counseling for lifestyle modification. The Kappa statistic for inter-rater reliability for indicator eligibility was .75 and, given agreement on eligibility, the kappa statistic for the indicated care was .91.

### Definitions

86% of patients with a visit in the last 13 months of the study period had a blood pressure measured. Patients were included in the study if their provider noted a diagnosis of hypertension in their medical record or if they had BP readings greater than 140/90 on two occasions at least two weeks apart. However, patients were only classified as having a new diagnosis of hypertension if this was specifically noted in their medical records. Per JNC-VI, we defined Stage 1 hypertension as BP 140/90–159/99 and Stage 2 hypertension as BP > 160/100; we determined stage by averaging a patient's BP over the study period, regardless of notation of diagnosis or treatment regimen. We defined uncontrolled hypertension as a last systolic BP ≥ 140 or a last diastolic BP ≥ 90.

### Analytic methods

We determined both individual and overall quality scores for each patient. We calculated individual scores by determining a patient's eligibility for an indicator and whether s/he received the indicated care. We calculated overall quality scores by adding the number of instances in which a patient was eligible for and received the indicated care and dividing this number by the total number of instances for which s/he was eligible for the indicated care; this score was expressed as a percentage. We defined optimal quality as patients having received all of the indicated care for which they were eligible. We adjusted the scores for non-response using multivariate models that weighted respondents to be representative of the population from which they were drawn. We used the bootstrap method to directly estimate standard errors for all of the individual indicator scores [18]. Statistical comparisons at the bi-variate level were reported as t-tests for continuous variables and chi-square tests for categorical discontinuous variables. A multivariate regression model was constructed to explain BP control using clinical and demographic variables available from the medical record.

## Results

Of the 6,712 adult patients for whom we analyzed medical records1,953 (29%) had a diagnosis of hypertension noted in their medical record or repeated BP readings indicative of hypertension. Of these, 241 (12%) were newly diagnosed during the study period; 1,102 (57%) had hypertension noted in the medical record, but the diagnosis was not new; 610 (31%) had repeated BPs ≥ 140 systolic and/or 90 diastolic evidencing hypertension, but no diagnosis in the chart. The sample included 1,070 women (55%), 1,326 patients over age 50 (68%), and 918 (47%) patients with CAD, hyperlipidemia, or diabetes noted in their medical records. The average last systolic BP was 139.2 and the average last diastolic BP was 82.0. Of the 1,368 patients (70%) who were receiving pharmacological treatment for hypertension, 25% were prescribed beta-blockers, 37% diuretics, 30% calcium channel blockers, and 35% angiotensin converting enzyme inhibitors or angiotensin II receptor antagonists. Patients with a diagnosis of hypertension noted in their records were more likely to have received pharmacotherapy during the study period than those with elevated BP readings but no notation of a diagnosis (88% vs. 31%, p < .0001).

On average, patients were eligible for 3.8 indicators (range: 1–21 and visited their providers 13 times during the study period. Table 1 shows the performance of the 28 indicators among the sample. Indicator #1 had the largest number of eligible patients (n = 1,953); Indicator # 27 had the fewest number of eligible patients (n = 20). Indicator scores ranged from a low of 14% for Indicators #5 and #20 to a high of 94% for Indicator #24. Indicator #28 required a change in regimen in response to persistently elevated blood pressure; 80% of patients satisfied this requirement with a change in medication and 20% with repeated counseling on nonpharmacologic means of BP control. The mean patient overall quality score was 72%, and 57% of patients (1,113) received optimal quality of care (i.e., they received all recommended care for which they were eligible). The overall quality score was higher for patients with a diagnosis of hypertension noted in their chart (86% vs. 41%, p < .0001).

The last BP reading for 42% (825) of patients indicated good control (<140/90). Table 2 shows the proportion of patients with well-controlled BP who received optimal and sub-optimal care by subgroup. Overall, patients with optimal quality care were more likely to have their BP under control (44.9% vs. 35.1%, p < .0006). This held true for all age groups, and for men, but was not statistically significant for women. Patients with optimal quality scores who did not have diabetes, CAD, or hyperlipidemia were more likely to have their BP controlled than those with sub-optimal scores.

**Table 2:** Proportion of patients with well controlled BP by level of quality of care received

**Group**	**N**	**% <140/90 (Standard error)**		**P-value**
			
		**Optimal quality**	**Sub-optimal quality**	
All	1,953	44.9 (2.0)	35.1 (2.0)	.0006
Female	1,070	41.2 (2.5)	37.5 (2.7)	.3209
Male	883	48.9 (2.8)	32.7 (3.0)	.0001
Age <= 50	627	49.5 (3.4)	39.1 (3.0)	.0234
Age > 50	1,326	43.4 (2.4)	32.2 (2.7)	.0020
Diabetes	372	48.5 (3.7)	43.9 (5.4)	.4797
No diabetes	1,581	44.0 (2.2)	33.5 (2.2)	.0008
CAD	337	43.6 (3.9)	38.4 (6.3)	.4835
No CAD	1,616	45.3 (2.3)	34.8 (2.1)	.0007
Hyperlipidemia	604	47.4 (3.3)	39.7 (4.2)	.1496
No hyperlipidemia	1,349	43.5 (2.4)	33.7 (2.3)	.0033
CAD, DM, or hyperlipidemia	918	46.2 (2.7)	41.9 (3.5)	.3237
Smoker	352	47.6 (4.7)	37.9 (4.1)	.1218
Nonsmoker	1,601	44.4 (2.1)	34.3 (2.4)	.0015

A logistic regression (not shown) revealed that the relationship between higher quality scores and BP control persisted when controlling for number of physician visits, baseline blood pressure control, gender, age, smoking status, and presence of CAD, diabetes, or hyperlipidemia. Other than quality, only baseline BP control was significantly associated with BP control at the end of the study. The relationship between quality and BP control also persisted when we restricted the study sample to those with at least two BP readings 12 months apart (n = 1,321). Likewise, there were no changes in direction or significance when we controlled for BP at 160/100 or used the continuous measures of last systolic or diastolic BP reading as the outcome.

**Figure 1:**
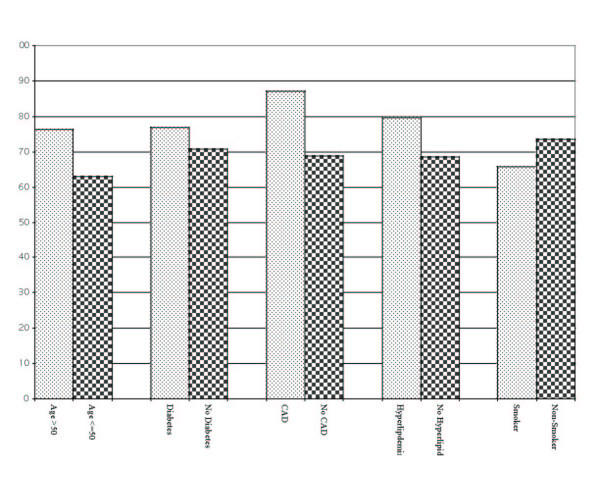
Overall process quality scores by demographic and clinical subgroups

Figure 1 shows the relationship between quality scores and various demographic and clinical characteristics. There was no significant difference between quality for men and women; however, patients over age 50 received better care than their younger cohorts (76% vs. 63%, p = <.0001), as did patients with diabetes (77% vs. 71%, p = .0038), CAD (87% vs. 69%, p = <.0001), or hyperlipidemia (80% vs. 68%, p = <.0001). In addition, smoking was associated with lower quality of care (66% vs. 73%, p = .0005).

## Discussion

Our data suggest that quality of care for hypertensive patients falls short of the ideal. Overall, patients received about 72% of recommended essential care processes, and 77% of patients with persistently elevated blood pressure had some change in therapy noted in the medical record over the course of two years. Though still concerning, these rates are higher than in some previous studies in more limited populations [9,19]. Some of the differences may be due to differences in the study population, and some may be due to more liberal definitions of what constitutes a change in therapy. We allowed longer time periods for the change to occur, and counted switching to different but possibly equipotent regimens and repeated nonpharmacologic interventions as therapeutic changes.

Patients with additional risk factors for cardiovascular events were more likely to receive recommended care, suggesting that providers may be targeting patients at highest risk for hypertensive complications. Alternatively, these patients may have had better BP control because they were already engaged in treatment for other conditions. Smoking was the exception to this pattern. Smokers received lower quality of care even though they are at higher risk for cardiac complications from hypertension. This is particularly surprising because cigarette smoking is the only measured risk factor that directly and immediately affects BP, though its effect on long-term hypertension is unclear [20]. Further research is needed to identify possible explanations, but we hypothesize that physicians provide lower quality of care to smokers because they perceive them as unwilling to participate in their own care or less likely to comply with medical therapy.

As in previous studies of hypertensives who have visited their physician, just under half of hypertensive patients had controlled BP. However, patients who received optimal care (i.e., they received all indicated care for which they were eligible) were more likely to have controlled BP at the end of the study, supporting the hypothesis that a broad range of hypertensive process quality is associated with better intermediate outcomes. The study was not powered to analyze which specific subcomponents of hypertensive care were most associated with BP control. However, because controlled hypertension is correlated with fewer myocardial infarctions and cerebrovascular accidents, it also seems likely that better overall care processes would eventually be associated with fewer complications from hypertensive care, although we could not directly test for this.

This process-outcome relationship between hypertensive care and BP control was observed in almost all subgroups, but differences in quality were statistically significant primarily in patients without other cardiac risk factors [4]. Unfortunately, our study could not detect small differences in the relationship between quality of care and BP control within cardiac risk factor subgroups, and this is likely to partially explain this finding. However, for patients with CAD or diabetes, another potential explanation could be a "ceiling effect": diabetic patients received 77% of recommended care and patients with CAD received 87%, leaving little variation in quality to explain differences in BP control.

In addition to poor process quality, there are other possible explanations for the high rates of uncontrolled BP observed in this study. Even among patients with at least one medical visit in the last two years, impaired access to care might be a factor, and this problem was likely worse in the 863 patients with no visits during the study period who were not included in the analysis [4]. Among those included in the study, however, impaired access was likely a small factor since the average number of provider visits in the two-year study period was 14 for patients with controlled BP and 12 for those with uncontrolled BP. In addition, hypertensive quality of care is no better in Canada despite the presence of universal health insurance in Canada and high prevalence of access problems in the U.S. [21], suggesting that sub-optimal clinical care processes are as important as access in explaining our results. Patient noncompliance has also long been recognized as an important predictor of poor BP control, but we did not measure it directly [22].

Our study is the first to examine the relationship between the quality of hypertensive care and outcomes among a national population; however, some study limitations exist beyond those inherent in retrospective medical record reviews. First, in constructing the indicators, our expert panel was instructed to rate indicators highly only if documentation of the process in the medical record was common and required for good quality care. Nonetheless, it is possible that documentation differences rather than true quality differences explain some of the observed variations in process quality or outcomes. A previous study found that differences in process scores were 10% lower for medical record abstraction compared to standardized patients with audiotapes of encounters [23]; thus, our study may underestimate actual quality of care by that amount. Second, we chose 140/90 as the threshold for poor BP control based on expert guidelines in force at the time of the study, but this threshold could potentially affect our results. However, sensitivity analyses using a higher threshold (160/100) and continuous distribution did not change our results. Later guidelines have suggested lower thresholds for diabetics; our findings remained consistent when we used 130/85 for this population. Third, providers' failure to measure the blood pressure could have caused us to erroneously exclude some patients from the analysis, though the vast majority of the studied patients had regular blood pressure measurement. Fourth, our response rate could have biased our results, particularly our estimates of overall quality. To account for this problem, we used standard techniques to adjust for non-response. Moreover, responders were more likely to be older and have chronic conditions than non-responders. Since we found higher quality of care among these subgroups, it is likely that our estimates represent upper bounds. Finally, our study does not take into account the lower threshold for pre-hypertension that was recently published in JNC-VII [5], since the care we evaluated was delivered prior to this recommendation. Future research will need to investigate whether the new standards are associated with better control.

Quality assessment in hypertension is only useful if it is linked with efforts to improve care. There is some evidence that quality improvement (QI) programs can lead to BP control among hypertensive patients. Godley has recently showed that a QI program in a group-model managed care organization increased BP control from 37% to 49% [24]. Our results can help providers focus on the processes most likely to improve control and avoid adverse outcomes.

## Conclusions

We found that the average hypertensive patient in the United States did not receive one in four essential care processes, and those with sub-optimal care had worse BP control. Our data indicate that improvements in processes of care may lead to better outcomes. While it appears that providers appropriately focus attention on patients with additional cardiovascular risk factors, they are under-treating other hypertensive patients who (as previous research would suggest) are nonetheless at increased risk for the same adverse outcomes. Future research should test if the application of routine measurement of hypertensive process quality in provider groups or plans leads to improved processes and BP control.

## Competing interests

Drs. Asch, McGlynn, Adams, and Kerr declare they have no competing interests. Ms. Hiatt, Hicks, and DeCristofaro declare they have no competing interests. Drs. Chen and LaPuerta are employees and own shares of Bristol-Myers Squibb Company.

## Authors' contributions

SA and BM served as principal investigators of this study and were involved in all aspects, as were EK and LH. PL and RC helped arrange partial funding and were involved in the analysis and writing of the manuscript. JA led the analysis, AD and JH were involved in supervising the data collection and participated in the analysis.

## Pre-publication history

The pre-publication history for this paper can be accessed here:


